# Four-year Survival (and Counting) after Stereotactic Radiosurgery to Nearly 100 Brain Metastases: A Case Report

**DOI:** 10.7759/cureus.2103

**Published:** 2018-01-23

**Authors:** Robin Saiki, Amanda Brill, Robert E Breeze

**Affiliations:** 1 Neurosurgery, University of Colorado

**Keywords:** keywords brain metastases, stereotactic radiosurgery, gamma knife, melanoma, multiple brain metastases

## Abstract

Brain metastases are a relatively common consequence of Stage IV melanoma. Historically, patients with brain metastases fare poorly, with median survival of only weeks to months. Patients with multiple metastases in the brain have often been overlooked in the literature, with the focus being placed more on patients with only a small number of metastases.

The authors present a case of a 42-year-old man with a total of 98 brain metastases treated over several Gamma Knife sessions. He is nearly five years out from his initial treatment.

This case highlights the fact that there is a large amount of variability in survival after diagnosis with brain metastases. Selection for treatment should be based on the clinical picture and clinicians should take care to avoid selection bias in this population.

## Introduction

Brain metastases are a common and devastating result of metastatic malignant melanoma. Melanoma is the third most common malignancy to metastasize to the brain, after breast and lung cancers [1]. Despite many improvements in the treatment of this disease, controversy persists regarding the optimal manner in which to treat intracranial metastases arising from malignant melanoma [2]. In fact, much of the literature reveals a bias against aggressive treatment of patients with more than a small number (three or less) of brain metastases, highlighting a somewhat nihilistic attitude toward this population [3].

When melanoma spreads to the central nervous system (CNS), the disease carries an exceptionally high rate of morbidity and mortality. Metastatic melanoma has a particularly high rate of spread to the intracranial compartment, with up to 40% of patients affected. This leads to a neurologic death in up to 95% of this group [4]. Median survival rates for patients with untreated brain metastases are only weeks, and patients who undergo treatment of their intracranial disease do not fare much better, with survival rates of typically less than one year [3].

## Case presentation

A 42-year-old male was referred to our neurosurgery clinic for management of newly discovered brain metastases secondary to malignant melanoma. He was initially diagnosed with melanoma in early 2012 after he noted a lesion on his scalp and developed local lymphadenopathy. He underwent biopsy followed by wide excision and lymph node dissection which confirmed a diagnosis of metastatic melanoma. His tumor was found to carry the BRAF V600E mutation. He progressed quickly, with diffuse metastatic disease throughout the body by August 2012.

**Figure 1:**
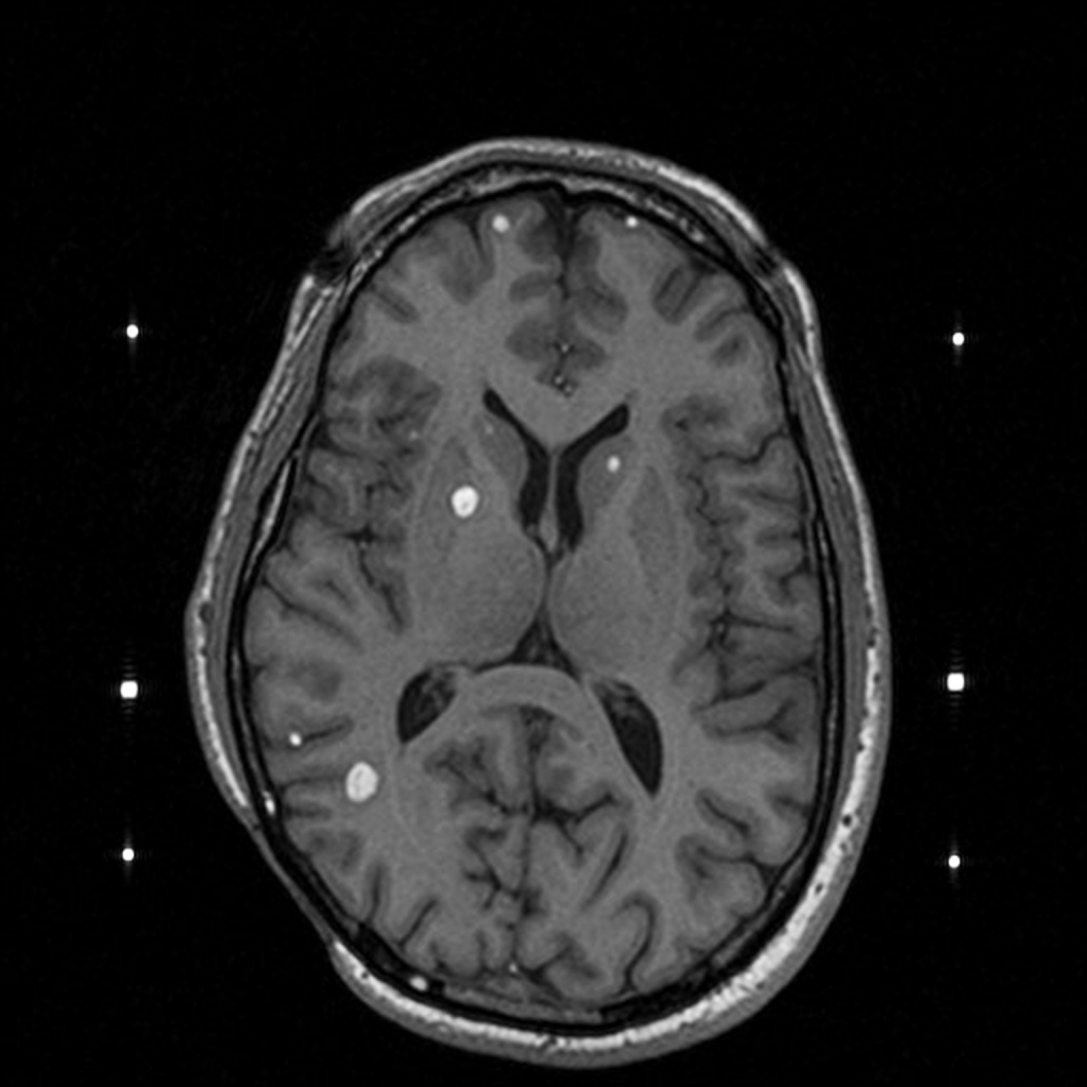
Axial imaging at the time of one of the patient's Gamma Knife treatments.

Shortly after his diagnosis with metastatic disease, he was placed on a trial and treated with PD-1 ligand antibody and vemurafenib. Despite this therapy, in February of 2013, a surveillance magnetic resonance imaging (MRI) of the brain demonstrated approximately 20 new brain metastases (see Figure 1). His systemic therapy was switched to ipilimumab at that time and he received four doses from early March to May of 2013. Due to the fact that the patient remained quite well from a clinical standpoint (Karnofsky = 100) and was completely asymptomatic from his brain metastases, we recommended that the patient undergo stereotactic radiosurgery (SRS) with the Gamma Knife. We elected to treat him in stages and his first treatment was in late March of 2013. At the time of every follow-up Gamma Knife scan, several new metastases were noted. In all, the patient underwent seven Gamma Knife procedures to treat a total of 98 brain metastases. His last treatment for intracranial disease was in May of 2015 at which time a lesion that was previously treated with SRS twice was surgically resected due to continued radiographic progression. Since that time, he has had no progression of his intracranial disease.

Throughout all of the intracranial treatments, the patient has enjoyed a good clinical status with a Karnofsky score of 100 and has been able to continue his full-time position as a software engineer.

## Discussion

This case is notable for several reasons. First, and most conspicuously, the number of lesions treated in this patient. While SRS is increasingly being utilized in the treatment of brain metastases, early studies in this arena suggested that Whole Brain Radiation Therapy (WBRT) be considered the mainstay for patients with more than four metastases [5]. In 2014, Yamamoto, et al. published data that implied that there was little difference in survival between groups of patients treated with SRS whether they had two to four metastases or five to 10 metastases [6]. This raised the question that is still being explored: is there a threshold beyond which SRS should not be considered as a treatment modality? Published literature on the treatment of more than 10 brain metastases with SRS remains limited.

Second, at nearly five years after his first diagnosis of intracranial disease, this patient’s survival is far above and beyond most published reports related to the length of survival in brain metastases from metastatic melanoma. In 2011, the University of Pittsburgh examined 333 patients with melanoma brain metastases treated with Gamma Knife (GK). Stereotactic radiosurgery with the GK was utilized as the primary treatment in 65% of this group, with 32% of subjects having four or more metastases in the brain. Median survival was 5.6 months after the time of radiosurgery [7]. In 2017, a retrospective study of 823 melanoma patients with brain metastases revealed a median overall survival of 9.8 months. The purpose of the study was to refine and modernize the Graded Prognostic Assessment for melanoma. More than half of this cohort received SRS alone as the means of treatment of their intracranial disease, but systemic therapy utilized varied widely by type, timing, and sequence in reference to diagnosis of brain metastases [8]. It is worth noting that our patient's score utilizing this tool was 2 which yields a predicted survival of 8.3 months with a range of 3.9 to 18.2 months. As previously noted, our patient's length of survival at this time is 59 months.

Since the 1990s, the University of Colorado has adopted more aggressive treatment guidelines for patients with brain metastases secondary to malignant melanoma due to suspicions that selection bias was influencing the length of survival in this group [9]. In short, all patients with malignant melanoma who have lesions consistent with brain metastases are considered for treatment if their clinical status is deemed adequate as defined by a Karnofsky Performance Score (KPS) of greater than or equal to 70. These patients are offered surgical resection of symptomatic lesions and GK radiosurgery of asymptomatic lesions without regard to total number or presence of extra-cranial disease. If the total number of asymptomatic lesions is greater than 10, treatment is divided into two or more stages, such that any given GK radiosurgery session is no more than three to four hours in length. Whole brain radiation is reserved for those patients with classic carcinomatous meningitis in whom palliative care or transition to hospice is felt to be warranted based on discussion with the patient’s oncologist. Following the initial treatment, surveillance MR scans are obtained at six- to eight-week intervals and additional treatment is offered to any patient still enjoying a good clinical status.

Anecdotally, the authors have noted that while brain metastases often recede after initiation of some systemic therapies, they enlarge at the six- to 12-month range. For this reason, it is our practice is to treat even those lesions that initially appear to be responding to systemic treatments.

## Conclusions

The authors recognize that this patient’s excellent outcome is a statistical outlier that is likely a result of a combination of the types of systemic therapies he received and SRS. However, the point to be emphasized is that there is a great degree of variability in survival among patients with brain metastases from malignant melanoma. In the past, this patient would have been automatically relegated to hospice care given his burden of intracranial disease. Moving forward, clinicians should consider developing and adopting more aggressive protocols for treatment of intracranial melanoma in this era of immunotherapy and targeted therapy.
